# Detecting modules in biological networks by edge weight clustering and entropy significance

**DOI:** 10.3389/fgene.2015.00265

**Published:** 2015-08-27

**Authors:** Paola Lecca, Angela Re

**Affiliations:** ^1^Centre for Integrative Biology, University of TrentoItaly; ^2^Laboratory of Translational Genomics, Centre for Integrative Biology, University of TrentoTrento, Italy

**Keywords:** protein–protein network, weighted network, node weight, edge weight, clustering, connected component, entropy

## Abstract

Detection of the modular structure of biological networks is of interest to researchers adopting a systems perspective for the analysis of omics data. Computational systems biology has provided a rich array of methods for network clustering. To date, the majority of approaches address this task through a network node classification based on topological or external quantifiable properties of network nodes. Conversely, numerical properties of network edges are underused, even though the information content which can be associated with network edges has augmented due to steady advances in molecular biology technology over the last decade. Properly accounting for network edges in the development of clustering approaches can become crucial to improve quantitative interpretation of omics data, finally resulting in more biologically plausible models. In this study, we present a novel technique for network module detection, named WG-Cluster (Weighted Graph CLUSTERing). WG-Cluster's notable features, compared to current approaches, lie in: (1) the simultaneous exploitation of network node and edge weights to improve the biological interpretability of the connected components detected, (2) the assessment of their statistical significance, and (3) the identification of emerging topological properties in the detected connected components. WG-Cluster utilizes three major steps: (i) an unsupervised version of k-means edge-based algorithm detects sub-graphs with similar edge weights, (ii) a fast-greedy algorithm detects connected components which are then scored and selected according to the statistical significance of their scores, and (iii) an analysis of the convolution between sub-graph mean edge weight and connected component score provides a summarizing view of the connected components. WG-Cluster can be applied to directed and undirected networks of different types of interacting entities and scales up to large omics data sets. Here, we show that WG-Cluster can be successfully used in the differential analysis of physical protein–protein interaction (PPI) networks. Specifically, applying WG-Cluster to a PPI network weighted by measurements of differential gene expression permits to explore the changes in network topology under two distinct (normal vs. tumor) conditions. WG-Cluster code is available at https://sites.google.com/site/paolaleccapersonalpage/.

## 1. Introduction

With biology increasingly becoming a data-rich field, objectives of systems biology research include organizing molecular interactions as networks and characterizing their structure, dynamics, and controllability. Since the turn of the century, high-throughput interaction mapping has emerged as an extremely useful approach to identify the constituents and connections of these networks. For instance the systematic identification of pairwise protein interactions (Rual et al., 2005; Petschnigg et al., 2014) or protein associations into complexes (Havugimana et al., 2012) has been enormously valuable both for understanding the function of individual proteins and for elucidating the organizing principles of the cellular physical architecture. Additional types of interactions have been charted including protein-DNA (Chen et al., 2015), protein-RNA (Moore et al., 2014; Re et al., 2014) and kinase-substrate (Linding et al., 2007; Varjosalo et al., 2013) interactions. Many of the molecular interaction data generated find their way into database resources that are available online (Turner et al., 2010; Horn et al., 2014; Orchard et al., 2014). The ability to generate, process and integrate omics data is instrumental to increasingly faithful reconstructions of the information flow in biological systems. In this vein, the conceptualization of biological systems as networks and the subsequent reconstruction of their modular organization acquire great interest (Barabási and Oltvai, 2004; Barabási et al., 2011; Ideker and Krogan, 2012). The notion of a module refers to a discrete entity whose constituent elements are similar in some quantifiable (e.g., chemical, physical, or functional) property and/or in the profile of their relationships. Biology displays many examples of modules which generally accomplish relatively separable functions such as nucleic acid synthesis, DNA replication, mitotic spindle assembly and protein degradation (Hartwell et al., 1999; Barabási and Oltvai, 2004).

In recent years, a rich collection of computational approaches has emerged for module detection in weighted networks, where weights can be constrained by topological or alternative numerical properties of nodes (for example, node molecular activity extracted from transcriptomics profiling) and edges (for example, edge confidence). Aside from weight assignment either to nodes (Ideker et al., 2002; Bader and Hogue, 2003) or to edges (Tanay et al., 2004; Liu et al., 2009; Pandey et al., 2014), clustering algorithms differ in the procedures for finding modules including, for example, simulated annealing (Ideker et al., 2002), greedy (Chuang et al., 2007; Nacu et al., 2007), genetic (Klammer et al., 2007), and network propagation (Vandin et al., 2011; TCGA Research Network, 2013) algorithms. Despite all of this exciting research in network clustering, some limitations stand out as remarkable. First, processing tens of thousands of nodes and the edges among them is hard to accomplish in fast timescales. Second, albeit equally interesting properties, it remains unclear how to meaningfully account for both node and edge weights in a module detection procedure.

Here, we present a novel algorithm for modular structure detection, named WG-Cluster (Weighted Graph CLUSTERing), which seeks to address previous shortcomings to detect modules. Within WG-Cluster, a module is defined as a connected component where nodes are characterized by homogeneous weights and are connected by edges of homogeneous weights. To this aim, WG-Cluster combines an edge-based network clustering with a fast-gready algorithm. The treatment of network edge weights within WG-Cluster represents a novelty compared to most clustering algorithms since, by the initial edge-based network clustering, network edge weights underlie the subsequent detection and prioritization of the connected components. Furthermore, the procedural choice adopted by WG-Cluster permits to obtain modules, homogeneous not only in node weights but also in edge weights, without discernible additional cost in computational efficiency. Module prioritization can become particularly useful in applications related to differential network analysis where the primary goal is to identify modules changing across different conditions. Finally, it is worth mentioning here also the introduction of a measure of the significance of the returned connected components which is based on node weights. WG-Cluster is here applied for the analysis of a differential network, i.e., a network where node and edge weights are defined by the changes observed in node and edge numerical properties between two conditions. Differential network analysis is useful to tackle the dynamic nature of molecular interactions, for instance as a consequence of environmental shifts. Computational integration of a network with molecular profiles acquired in different contexts has shown a popular approach to extract context-dependent responsive modules, which mark strikingly changed regions of the network. The input network for the current WG-Cluster application is a differential network, which was obtained by integrating a physical protein–protein interaction (PPI) network with changes in gene expression between a normal and tumor conditions. Our analysis showed that WG-Cluster is useful for comprehensively analysing the quantitative changes affecting nodes or interactions in the network and for recognizing modules which link to functional properties.

## 2. Materials and methods

### 2.1. Data description and pre-processing

We gathered multi-assay omics data to define the weighted network which is the primary input to WG-cluster. We collected PPIs from the open-access IntAct database which adopts a merging algorithm and a scoring system to provide richly annotated molecular interaction data. IntAct PPIs are described in the controlled vocabulary specified by the Proteomics Standards Initiative for Molecular Interaction (PSI-MI) data (Hermjakob et al., 2004) and adhere to the guidelines (Orchard et al., 2007) about the Minimum Information required for reporting a Molecular Interaction Experiment, which were supplied by the International Molecular Exchange (IMEx) consortium. PPIs involving human protein entities were selected and downloaded along with their confidence scores. Protein identifiers defined by the Universal Protein Resource (Uniprot) protein accessions (http://www.uniprot.org/) were mapped to gene identifiers defined by the HUGO Gene Nomenclature Committee (HGNC) gene symbols (http://www.genenames.org/). We next integrated the IntAct PPIs with tumor-dependent changes in messenger RNA (mRNA) expression profiles. Processed gene expression data related to colon adenocarcinoma were downloaded from The Cancer Genome Atlas (TCGA) (http://cancergenome.nih.gov/). mRNA profiles were generated from 155 tumor and 19 normal tissue samples. Processed data were lowess normalized and collapsed by gene symbol (log_2_ scale). A differential co-expression score was computed for each gene pair, by subtracting the pairwise Pearson's correlation coefficient in the tumor condition from that in the normal condition. Next, the IntAct PPI confidence scores were multiplied by the differential co-expression scores to estimate the change in the interaction strength resulting from the differential co-expression of the mRNAs encoding the interacting proteins. The product between the IntAct score and the differential co-expression score defines the final weight of an edge in the differential network. The weight of a node in the differential network was obtained by computing the ratio between the average values of mRNA expression across samples in the normal and tumor conditions (mRNA fold change). This differential network, where both nodes and edges were weighted, was the primary input to the WG-Cluster algorithm.

### 2.2. WG-cluster

**Figure 1:**
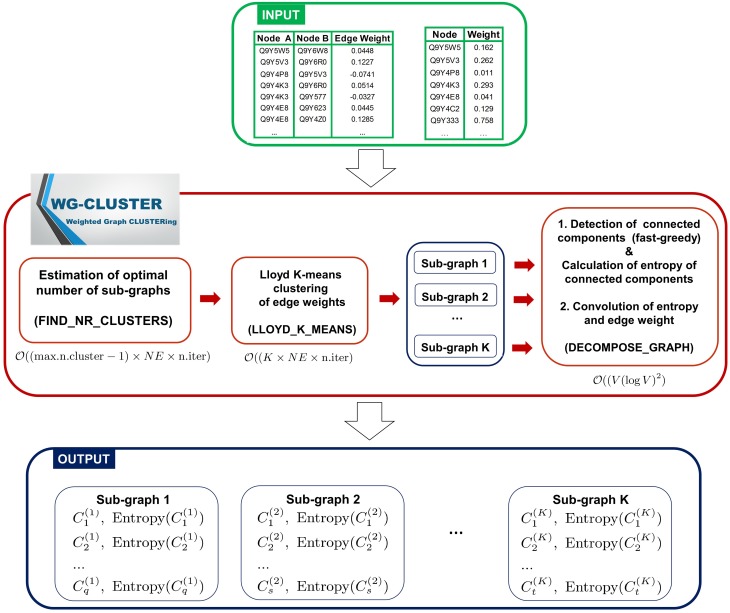
**Algorithmic modules of WG-Cluster**. WG-Cluster takes as input the SIF file of the network edges and a text file reporting node labels in the first column and node weights in the second one. If the second file is not available, WG-Cluster by default assigns an equal weight to all nodes. WG-Cluster implements three computational modules: (i) an unsupervised version of the K-means algorithm identifies sub-graphs with similar edge weights, (ii) a fast-greedy algorithm detects the connected components of each sub-graph utilizing similarity in node topological properties, (iii) the estimates of the convolution of connected component entropy and sub-graph mean edge weight guide the selection of significant connected components representative of global trends in the network. Complexity of modules for estimating the optimal number of sub-graphs and for running the Lloyd's K-means is linear in the number of edges *NE* and number of iterations; the complexity of the module for detecting connected components is Ҩ(*V*(log*V*)^2^), where *V* is the number of vertices.

The WG-Cluster algorithm is implemented in R (R software available at http://www.r-project.org), which provides one of the most widely used, most flexible and mature open source environments. For the most computationally intense tasks WG-Cluster employs built-in R functions implemented as a C(++) or Fortran code, that are optimized and faster than functions coded in R from scratch. The input data consist of the network edges reported in Simple Interaction File (SIF) format (Cytoscape, 2015) and of node weights reported in tabular format (node, weight). The algorithm sequentially executes three computational modules. First, it estimates the optimal number of clusters (sub-graphs) that split up the graph (i.e., network) and executes a Lloyd's K-means clustering (Du et al., 2006) of the edge weights to detect sub-graphs with edges of similar weights. Second, a fast-greedy modularity optimization procedure (Clauset et al., 2004) finds (if any) the connected components (i.e., modules) in each sub-graph. An entropy score is computed for each connected component and is used as a measure of the statistical significance of the connected component. Finally, an analysis of the convolution between sub-graph mean edge weight and connected component entropy allows for a summarizing view of both properties in the detected connected components (Figure 1). In the following, we give the details about each computational module of WG-Cluster. Hereafter, we will denote with *V* the number of vertices and with *NE* the number of edges in the input graph.

#### 2.2.1. Detection of sub-graphs

The optimal number of sub-graphs which partition the input graph is estimated by minimizing the total within-clusters sum of squares (WCSS) obtained with a K-means procedure. For a set of edge weights **w** = (*w*_1_, *w*_2_, …, *w*_*NE*_), K-means clustering tries to find a set of *K* sub-graphs *S* = (*S*_1_, *S*_2_, …, *S*_*K*_) that is a solution to the minimization problem:

$$\begin{array}{l} WCSS=\overset{K}{\underset{i=1}{\sum }}\underset{\text{w}\in S_{i}}{\sum }||\text{w}-\mu_{i}|{|}^{2} \end{array}$$

where μ_*i*_ is the mean of the edge weights **w** in the sub-graph *S*_*i*_.

An elbow in the curve interpolating the points (*n*_sub-graphs_, WCSS) suggests the appropriate number of sub-graphs *n*_optimal_. In our implementation, *n*_optimal_ is estimated as the minimum value of *n*_clusters_ at which the first derivative of WCSS w.r.t. *n*_sub-graphs_ is null within a tolerance 0 < ϵ ≪ 1, i.e.,

$$\left|\frac{d\;WCSS}{dn_{\text{sub-graphs}}}\right|\leq \epsilon .$$

The first derivative of the curve (*n*_sub-graphs_, WCSS) is calculated by the Stineman algorithm (Johannesson and Bjornsson, 2012). Algorithm 1 reports the pseudo-code of the first module of WG-Cluster.

**Algorithm 1:** Compute the optimal number of sub-graphs *K*

1: **procedure** FIND_NR_SUB_GRAPHS(edge.weights, max.n.sub.graphs, seed)
2:
3: *NE* ← Number of edges of the graph
4:
5: **1. Calculate the within-cluster sum of squares (wcss) via a K-means solution**.
6:
7: wcss[1] ←(NE - 1) × Variance(edge.weights)
8:
9: set.seed(seed)
10: **for** (i in 2:max.n.sub.graphs) **do**
11: wcss[i] ← $\sum_{1}^{i}$ (K-means(edge.weights, centroids = i))
12: **end for**
13:
14:
15: **2 Estimate** *d WCSS*∕*dn*.*sub*.*graphs* **with the Stineman algorithm**.
16:
17: n.sub.graphs ← 1:max.n.sub.graphs
18: wcss.derivative ← Stineman.derivative(n.sub.graphs, wcss)
19:
20: **3. Set a tolerance value**.
21:
22: tolerance ← ϵ
23:
24: **4. Find the first local minimum of** *d WCSS*∕*dn*.*sub*.*graphs*
25:
26: wcss.derivative.null ← {−ϵ ≤ wcss.derivative ≤ ϵ }
27: K ← wcss.derivative.null[1]
28:
29: **5. Return the optimal number of sub-graphs** *K*. Return *K*
30:
31: **end procedure**

The problem of WCSS minimization is known to be NP-hard, implying long running times, that can become unacceptable in case of biological networks with thousands of nodes and tens of thousands of edges. Furthermore, if the input data do not have a strong clustering structure, the procedure may not converge. For this reason, WG-Cluster adopts the Lloyd's algorithm whose complexity is linear in the number of edges and number of sub-graphs, and is recommended in case of data poorly clustered (Du et al., 2006). Algorithm 2 presents the pseudo-code of the Lloyd's K-means. Those iterations are repeated until the centroids stop changing, within a tolerance quantified by the parameter threshold (see the pseudo-code 2).

**Algorithm 2:** Lloyd's K-means algorithm

1: **procedure** LLOYD_K_MEANS(edge.weights, K, distance)
2:
3: **1. Randomly choose** *K* **items from the edge weights vector and use these as the initial means**.
4:
5: **2. Iterations of assignments and centroid recalculation**.
6: **while** distance(centroids, edge.weights) > threshold **do**
7: a. Assign edge weights to the centroids
8: **for** i ≤*NE* **do**
9: Assign edge.weights[i] to closest sub-graph according to the distance measure.
10: **end for**
11: b. Recalculate centroids.
12: **end while**
13: **end procedure**

In Supplementary Material (Section 1.1) we present the exploratory analysis of other clustering approaches and the motivation of the choice of the K-means algorithm in WG-Cluster.

#### 2.2.2. Detection of connected components

**Figure 2:**
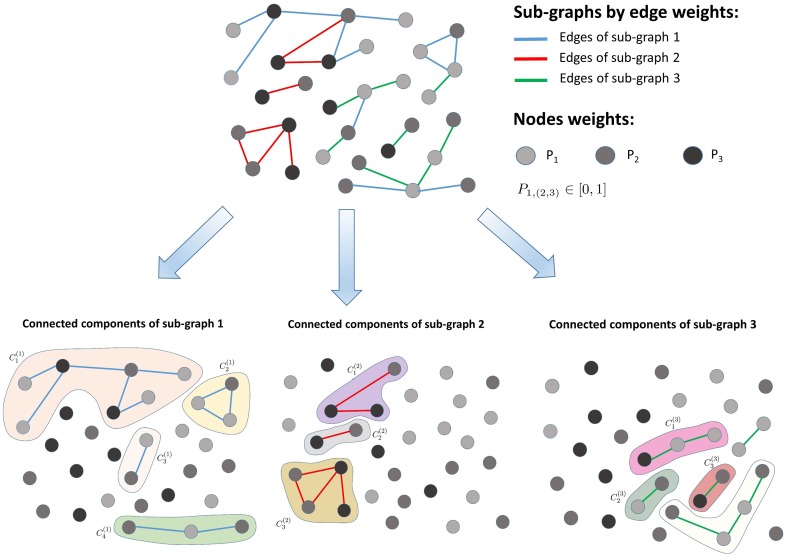
**Sub-graph decomposition into connected components**. The algorithm first clusters the input graph into sub-graphs consisting of similar edge weights and next detects the connected components present within each sub-graph.

Each sub-graph *S*_*i*_ (*i* = 1, …, *K*) returned by the K-means clustering is decomposed into connected components $C_{l}^{\left(i\right)}$ (with *l* = 1, 2, …, *L*_*i*_, where *L*_*i*_ is the number of connected components in the sub-graph *S*_*i*_) via a fast-greedy optimization procedure (Clauset et al., 2004), as illustrated in Figure 2. The entropy of each connected component is calculated as follows:

(1) $$\begin{array}{l} E_{C_{l}^{\left(i\right)}}=-\overset{N\left(C_{l}^{\left(i\right)}\right)}{\underset{j=1}{\sum }}\frac{p_{j}\log_{2}p_{j}}{d_{j}} \end{array}$$

where $N\left(C_{l}^{\left(i\right)}\right)$ is the number of nodes in the connected component $C_{l}^{\left(i\right)}$, *p*_*j*_ is the fold change of the expression level (from normal to tumor condition) of gene *j* (normalized between 0 and 1) and *d*_*j*_ is the sum of the weights of the edges adjacent to the node representing gene *j* (known as *node strength*). Denoting with *D*^(*j*)^ the number of nodes directly connected to node *j*, *d*_*j*_ is thus defined as

$$\begin{array}{l} d_{j}=\overset{D^{\left(j\right)}}{\underset{h=1}{\sum }}w_{jh}. \end{array}$$

where *w*_*jh*_ is the edge weight between the node *j* and its directly connected node *h*.

The entropy is used as a measure of significance of the connected components. In order to establish a threshold on the entropy significance, we generated for each connected component $C_{l}^{\left(i\right)}$ an ensemble of 100 random connected components with the same degree distribution of the reference connected component $C_{l}^{\left(i\right)}$.

A connected component is considered significant, and retained, if its entropy value is more than three standard deviations far from the mean entropy of the corresponding ensemble of random connected components. Let denote with $\left\{C_{l^{\prime }}^{\left(i^{\prime }\right)}\right\}$, where $l^{\prime }\in \left\{1,2,\ldots ,L_{i}^{\prime }\right\}$ with $L_{i}^{\prime }\leq L_{i}$, and *i*′∈{1, 2, …, *K*′} with *K*′ ≤ *K*.

#### 2.2.3. Convolution of mean edge weight and entropy

Both the connected component entropy and the mean weight of the edges of the sub-graph to which a connected component belongs are considered to classify the connected components.

The convolution of the entropy of selected connected components (*E*_selected_) with the mean edge weight *MW* of the sub-graphs to which they belong is performed as follows:

(2) $$\begin{array}{l} E_{\text{selected}}\left[h\right]*MW\left[h\right]=\underset{q}{\sum }E_{\text{selected}}\left[q\right]\cdot MW\left[q-h\right] \end{array}$$

where $E_{\text{selected}}=\left\{E_{C_{l^{\prime }}^{\left(i^{\prime }\right)}}\right\}$ is the vector of the entropies of the significant connected components, and $MW=\left\{\left(1∕NE^{\left(i^{\prime }\right)}\right)\overset{NE^{\left(i^{\prime }\right)}}{\underset{l=1}{\sum }}w_{l}\right\}$ is the mean edge weight of the sub-graph to which they belong.

The convolution in Equation (2) calculates the area overlap between the probability distributions of the entropy and of the mean edge weight as a function of the amount by which one of the distribution is translated. The area of the overlap of the two distribution measures the similarity between the entropy and mean edge weight distribution. The density of the convolution is a spectrum of the frequency of this similarity score and offers a way to classify the connected components by their membership to intervals of frequency corresponding to local maxima or minima of the convolution density. Maxima of the convolution density correspond to the most frequent values of similarity between entropy and mean edge weight, whereas local minima correspond to the least frequent values of similarity. Then, connected components can be classified according to the frequency of the convolution between their entropy and the mean edge weight of the sub-graph to which they belong. Algorithm 3 provides the steps of the pseudo-code implementing the procedure of detection and selection of significant connected components.

**Algorithm 3:** Detection and selection of connected components

1: **procedure** DECOMPOSE_GRAPH(sub-graphs, node.weights)
2:
3: **1. Detection of connected components and calculation of their entropy**.
4:
5: **for** (i in 1:K) **do**
6:
7: **a. Fast-greedy decomposition of the i**-*th* **sub-graph into connected components**.
8: connected.components[[i]] ← fast.greedy.decomposition(sub-graph[i])
9:
10: **b. Entropy calculation**.
11:
12: **for** (l in 1:*L*_*i*_) **do**
13: (**b.1**) **entropy of connected components with Equation (1)**.
14:
15: connected.components.entropy[l]
16: ← entropy(connected.components[[i]][l], node.weights)
17:
18: **(b.2) Generate an ensemble of random weighted Erdös-Renyi connected components**.
19:
20: **for** (v in 1:100) **do**
21: random.cc.component
22: ← erdos.renyi.graph(nr.of.nodes = $N(C_{l}^{\left(i\right)})$, nr.of.edges = $NE(C_{l}^{\left(i\right)}))$
23: edge.weights.random.cc.component ← Unif(0, 1)
24: node.weights.random.cc.component ← Unif(0, 1)
25: random.cc.entropies[v]
26: ← calculate.entropy(random.cc.component,node.weights.random.cc.component,edge. weights.random.cc.component)
27: **end for**
28:
29: **(b.3) Calculate the mean of the entropies of the ensemble of random connected components**.
30: random.cc.entropy[l]
31: ← calculate.mean.entropy(random.cc.entropies)
32: **(b.4) Select connected components**.
33: **if** cc.entropy[l] ∉ [-3 σ + random.cc.entropy[l] , +3 σ + random.cc.entropy[l] ] **then**
34: selected.connected.components ← $C_{l}^{\left(i\right)}$
35: **else**
36: discard $C_{l}^{\left(i\right)}$
37: **end if**
38: **end for**
39: **end for**
40:
41: **2. Convolution of Entropy (***E***) and mean edge weight (***MW***)**.
42:
43: **(a) Calculate the density of the convolution estimated by Equation (2)**.
44: density.of.convolution ← density(convolve (E, MW))
45: **(b) Detect the maxima of the convolution density**.
46: **(c) Select and return the connected components whose values of convolution of (E, MW) fall under convolution density maxima**.
47: **end procedure**

### 2.3. Functional analysis of connected components

Enrichment analysis based on the generic Gene Ontology (GO) slim (http://geneontology.org/), a cut-down version of the Gene Ontology annotations, was conducted for each retained connected component (hypergeometric test). GO enrichment *p*-values were transformed in Benjamini-Hochberg false discovery rate (FDR) values and retained at the significance level of 0.05.

## 3. Results

### 3.1. Performances on synthetic data

We evaluated the performances of WG-Cluster in processing Erdös-Rényi random graphs, consisting of 500 nodes and an increasing number of edges, in terms of user CPU running time.

**Figure 3:**
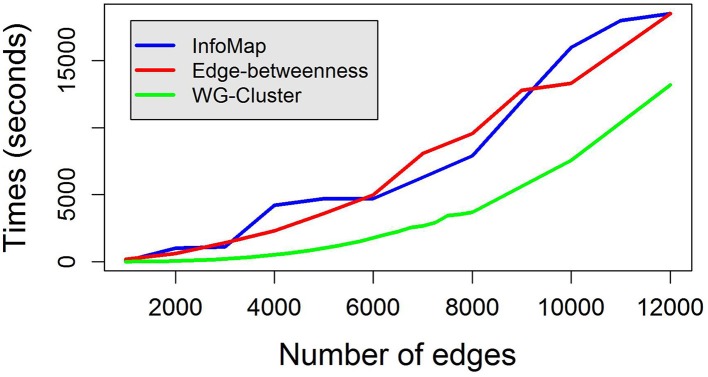
**Running times to cluster random weighted graphs with increasing number of edges**. WG-Cluster running time on a random weighted graph of 500 nodes and an increasing number of edges is compared with that achieved by the edge betweenness graph clustering algorithm (Girvan and Newman, 2001) and that of InfoMap (Rosvall and Bergstrom, 2008). Each algorithm was utilized in its R implementation on a desktop Windows 8.1 PC with a 3.1 GHz CPU. WG-Cluster ensured faster running time and a RAM usage inferior to 3Gb.

Edge weights were drawn from a uniform distribution between 0 and 1 and clustered in 10 groups. A uniform distribution between 0 and 1 was also used to obtain node weights. We compared WG-Cluster running times to the running times of three widely used deterministic hierarchical approaches to graph clustering: (i) edge betweenness based clustering, (ii) label propagation, and (iii) InfoMap, which were selected because they handle directed (as well as undirected) and weighted networks as WG-Cluster does (see Table 1 for a summary of the currently available deterministic clustering methods implemented in R). Non-deterministic clustering algorithms [e.g., Walktrap (Pons and Latapy, 2005), Spinglass (Reichardt and Bornholdt, 2006), and label propagation (Raghavan et al., 2007)] were left out of this comparative analysis since they require the determination of the number of runs needed to build a consensus partition. This parameter often depends on the topological structure of the graph and can remarkably affect the performances (that are usually satisfactory on single runs). We also excluded from the comparison the algorithms that do not handle the processing of undirected networks [e.g., Leading eigenvectors, (Newman, 2006)]. From this analysis, WG-Cluster showed to outperform the alternative algorithms (Figure 3).

**Table 1:** **Summary of widely used hierarchical methods for module detection**.

**Method**	**Type of graph**	**Weighted edges**	**Weighted nodes**
Edge-Betweenness (Girvan and Newman, 2001)	Directed and undirected	True	False
Fast-greedy (Clauset et al., 2004)	Directed and undirected	True	False
InfoMap (Rosvall and Bergstrom, 2008)	Directed and undirected	True	True

*“True” and “False” in the two last columns stand for “the method can process also” and “the method does not process,” respectively. For instance, edge-betweenness clustering method can process and take into account edge weights, but it does not handle information about node weights*.

In Supplementary Material, Section 1.2, we provide a more comprehensive analysis of the time complexity of WG-Cluster applied to random graphs of increasing number of edges and number of nodes.

Finally, further improvements in efficiency will be tested in the next version of WG-Cluster by the usage of recent libraries developed specifically to perform an optimized memory-efficient management of large datasets. The input/output and data rearrangement operations on large datasets are computationally time consuming, and their speeding is one of the main research topic engaging the developers of the majority of programming languages. R proposed two major solutions to optimize the efficiency of massive dataset processing (Kane and Emerson, 2013; Adler et al., 2014). Using these solutions, WG-Cluster could take advantage of the benefits of R (i.e., interactive data analysis and rich, flexible statistical programming environment), and, at the same time, of the benefit of C(++) language, i.e., an optimized memory-efficient management of big datasets.

### 3.2. Application

Biological systems are highly dynamical entities by depending on environment, tissue type, disease state or development. Nonetheless, relatively little effort has been spent in differential network analysis, i.e., the analysis of the changes occurring in a network in response to different conditions. Even though an increasing number of studies seek to analyse the dynamics of networks directly, through experimental mapping of networks across multiple conditions (Grossmann et al., 2015; Martin et al., 2015), a longstanding approach in differential network biology is to construct differential networks by integrating static (at standard laboratory conditions) molecular interaction networks (e.g., PPI networks) with changes observed in messenger RNA expression in different biological conditions (de Lichtenberg et al., 2005). The resulting differential network is a weighted network where node weights reflect the changes in mRNA expression levels and where edge weights reflect the changes in interaction strengths due to differential mRNA co-expression levels under the two conditions. It is worth noting that the strongest differential interactions are not necessarily the strongest ones in the static networks. Since both node and edge properties are deeply ingrained in the clustering procedure, WG-Cluster can provide a unique view of the differences in network topology between any two biological conditions.

As a proof-of-principle, we applied the WG-Cluster approach to analyse the differential PPI network that arises when tumor and normal conditions are contrasted. The current application focused on the colorectal cancer which stands among the most common cancers with more than 1.2 million new cases and about 600,000 deaths per year worldwide (Jemal et al., 2011). Messanger RNA expression data were obtained from The Cancer Genome Atlas which, importantly, provides samples from tumor tissues and from matched normal tissues. We acquired PPIs from the IntAct database because it provides a heuristic scoring system which relies on the available annotation evidences associated with an interacting pair of proteins. The differential network was constructed as follows: a node was weighted by the mRNA fold change and an edge was weighted by multiplying the IntAct PPI confidence score with the difference of the mRNA co-expression scores between the normal/tumor conditions. In the Supplementary Material (Sections 1.3 and 1.4), we provide a full description of this network edge weight model. Furthermore, we show that this network edge weighting approach leads to improved clustering quality compared to the classical approach which is based only on differential co-expression. The differential network consisted of 5569 nodes and 18,078 edges, out of which 8880 were strengthened and 9198 weakened in the tumor condition compared to the normal one.

**Figure 4:**
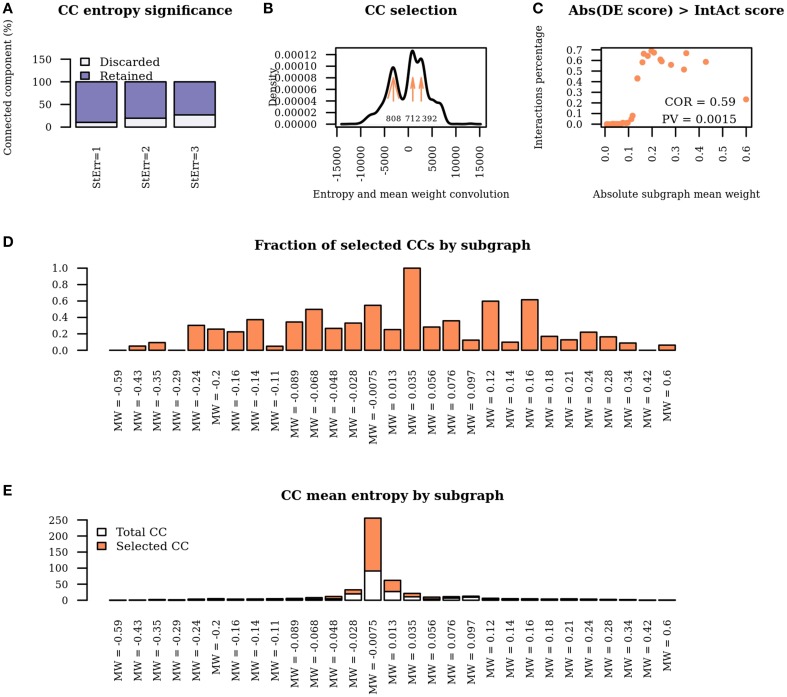
**Network properties of WG-Cluster reconstructed modules. (A)** Bar plot displaying the fraction of connected components which are discarded / retained according to the number of standard deviations of the entropy from the mean value of the distribution of entropy derived from randomized connected components. **(B)** Density plot of the convolution between the connected component entropy and mean edge weight of the respective sub-graph. Maximum points in the density plot are highlighted by arrows. The number at each arrow denotes the number of selected connected components, i.e., connected components whose entropy and mean edge weight correspond to convolution intervals at the maxima of the density plot. **(C)** Dot plot displaying the percentage of interactions yielding differential co-expression scores higher than IntAct scores as a function of subgraph mean edge weight. Scores are taken in absolute value. **(D)** Bar plot showing the fraction of connected components retained in each sub-graph. **(E)** Bar plot showing the mean entropy of connected components selected solely on the basis of entropy significance or on the basis of convolution analysis in each sub-graph.

Applying WG-cluster to the differential network detected 6215 connected components which were arranged in 29 sub-graphs of distinct mean edge weights. Upon connected components detection, WG-Cluster allows the estimation of the statistical significance of the entropy of each connected component by comparing the observed value against the distribution of entropies obtained from appropriately randomized connected components. The rate of connected component exclusion appeared stably moderate when we incremented the number of standard deviations from the expected entropy value; setting this number at three resulted in the exclusion of 26.87% of connected components (Figure 4A). Statistically significant connected components can be prioritized by any sort of network property such as mean edge weight of the sub-graph, or entropy or number of nodes by connected component. It is noteworthy that the numerical features associated with each connected component provide complementary information. For instance, correlation between mean edge weight and entropy values was not statistically significant (Spearman's coefficient = −0.02, *P* = 0.15). Since the mean weight of the edges in a sub-graph reflects the mean change in interactions strength and the entropy of a connected component reflects the mRNA expression changes, the observed lack of correlation is interesting because it is in agreement with previous data showing that the strongest differential interactions do not necessarily involve the strongest differential genes (Ideker and Krogan, 2012).

The last WG-Cluster step implements the convolution of the probability distribution of the connected component entropy with that of the sub-graph mean edge weight. This operation offers an appealing way to classify connected components in terms of both of those properties which, in our vision, are of equal interest. Since we were interested into obtaining a summarizing view of the network clustering, we selected the connected components yielding the most frequent convolution values (Figure 4B). We then interpreted those convolution values in terms of the corresponding sub-graph mean edge weight and connected component entropy values.

The number of the connected components obtained was found to increase in sub-graphs yielding lower mean edge weight (Figure 4D); conversely, no trend was detectable by analysing the mean entropy of the selected connected components resulting from each sub-graph (Figure 4E). Since the edge scores in the differential network result from the product of the IntAct scores with the differential co-expression scores, we verified that a low mean edge weight depended on low differential co-expression score, which resulted to be the case; indeed, the percentage of interactions where the differential co-expression score was higher than the IntAct score positively correlated with the sub-graph mean edge weight (Figure 4C).

**Figure 5:**
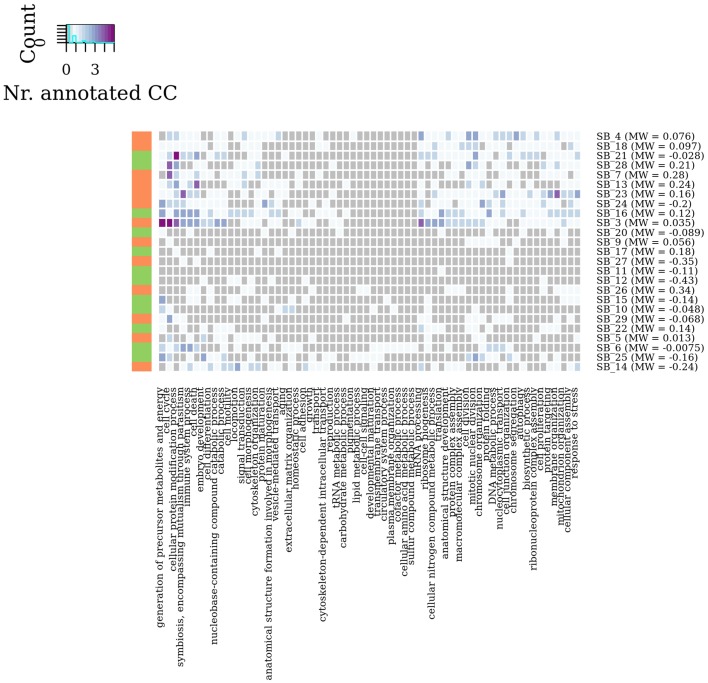
**Module enrichment in Gene Ontology categories**. Heat map showing in each sub-graph the number of connected components which resulted statistically significant enriched in GO Biological Process categories (adjusted *P*-value < 0.05). Vertical bar colors denote the sign of sub-graph mean edge weights.

In summary, by a general survey of WG-Cluster outcome, the majority of the detected connected components were found to consist of moderately changing interactions. More interestingly, the arrangement of the detected connected components by decreasing sub-graph mean edge weight (as shown in Figure 4D), which is inherent to WG-Cluster, streamlined the identification of connected components of markedly changing interactions. Those connected components, albeit limited in number, are undoubtedly the most interesting for unveiling the most striking changes in network topology between tumor/normal conditions (Figure 4D). Gene Ontology enrichment analysis was conducted to broadly assess the functional significance of module selection since exploring the fine details of specific modules is out of the scope of our study. This analysis showed that sub-graph clustering by mean edge weight broadly corresponded to a clustering of GO biological processes (Figure 5). Genes involved in cell cycle, cell death, mRNA processing and protein modification processes were found to be overrepresented in modules of weakened interactions in the tumor compared to the normal condition (sub-graph positive mean edge weight). On the other hand, genes acting in cell adhesion, extracellular matrix organization and cell-cell signaling resulted overrepresented in modules of interactions which were found strengthened in the tumor vs normal condition (sub-graph negative mean edge weight). It is reassuring that the GO categories overrepresented in the connected components were largely found in agreement with a previous survey of pathways consistently overrepresented in a large collection of signatures of differentially expressed genes of prognostic value in colorectal cancer (Lascorz et al., 2011). This case study showed that WG-Cluster allows shedding light into the network organization by fast and statistically robust module detection. In the context of a differential network analysis, it delivers emergent information about the quantitative changes of interaction strength and gene mRNA abundance between two conditions, and allows the user to pursue specific modules on the basis of any available biological rationale, including the extent of changes in interaction strength, the extent of mRNA fold change or the functional characterization of modules.

## 4. Discussion

Molecule interconnectivity in human cells is daunting with ~ 20,000 protein-coding genes and ~ 87,000 protein isoforms. Consequently, a network formalization of cellular processes is extremely useful to analyse the growing amount of data on many types of interactions, which include but are not limited to physical PPIs. A rich array of methods is currently available to detect network modular organization (Andreopoulos et al., 2009; Chen et al., 2014). Major limitations of most clustering methods, in very general terms, include the high computational cost and the inefficiency in exploiting the knowledge on edge strength (Toubiana et al., 2013). These aspects appear increasingly limiting in the light of the steady increase in the size of interaction maps and of the efforts to achieve interaction scoring standards (Villaveces et al., 2015). In this work, a new algorithm for network clustering has been developed that leverages existing information on both network nodes and edges to efficiently provide statistically significant modules. The detected modules are allowed to overlap, which reflects a common biological scenario, where, for instance, proteins can participate in multiple functions by participating to multiple functional modules. Within WG-Cluster, the connected components are homogeneous in terms both of node weights and of edge weights. We required homogeneity to extend to the numerical properties assigned both to network nodes and edges as both of them are expected to be biologically informative and useful to prioritize the study of the clustering results. To detect the modules, not only the reachability among nodes but also the homogeneity in the edges connecting the nodes has to be verified. To avoid the simultaneous verification of both requirements, which is highly time-consuming, WG-Cluster separates the two operations, firstly by identifying sub-graphs of homogeneous edge weights and, secondly, by detecting modules within each sub-graph. This procedural choice ensures, in an efficient way, connected components to be homogeneous in edge weights by construction. Furthermore, an entropy score is assigned to each connected component, which reflects the weights of nodes included in the connected component. The entropy score is utilized to measure the statistical significance of each component. Although not submitting node weights is allowed in WG-Cluster, it is worth noting that this choice invariably leads to entropy estimates which only depend on purely structural node properties. Therefore, partial input data limits the richness of information which could be made available by WG-Cluster. Finally, a convolution analysis of the entropy of the connected components with the mean edge weight of the sub-graphs was introduced to provide a global overview of the returned connected components and inform downstream analysis.

WG-Cluster is a method to cluster weighted networks into connected components, where nodes are homogeneous in their weights and are connected to each other by edges of homogeneous weights, and therefore WG-Cluster is suitable for many applications. A prominent applicative context is related to differential network analysis, which can discern cellular processes differently active under different conditions, such as with or without treatment by a pharmacological agent, with or without disease. Differential approaches have begun to drive considerable efforts in network biology, through the development of experimental assays to directly capture condition-specific networks (Ochoa and Beltrao, 2015) or through the integration of networks with condition-specific molecular profiles (Ideker et al., 2002; Jansen et al., 2002; Guo et al., 2007).

The case study presented here suggests WG-Cluster as a possible method for differential network analysis. A network of physical PPI interactions, which are scored utilizing community standards and are deposited in the IntAct database, was integrated with mRNA expression data acquired from colon adenocarcinoma tumor samples or from normal samples. Our integrative approach relied on the rationale that the strength of a protein–protein interaction depends on the extent of congruent protein levels and on their protein affinity. Under the assumption that protein expression can be approximated with mRNA expression and that the interaction score in IntAct reflects the interaction affinity, we specified nodes and edge weights of the differential network as follows. Node weights were defined by the mRNA level fold changes while edge weights were defined by the product of the IntAct scores with the differential mRNA co-expression scores between the two conditions. Applying WG-Cluster to the differential network permitted to prioritize modules in the PPI network representing regions of progressively decreasing changes between the tumor and normal conditions. Despite the fact that the majority of interactions changed moderately between the two conditions, the organization of the detection of weighted connected components by sub-graph, which is implemented in WG-Cluster, permitted to streamline the identification of modules of markedly changing interactions. Furthermore, it was possible to discern modules of interactions which get weakened or strengthened in the tumor compared to the normal condition. Interestingly, separating the modules by average increase or decrease in the strength of their interactions reflected also on their functional enrichment into distinct GO categories.

WG-Cluster is available as an open-source tool at https://sites.google.com/site/paolaleccapersonalpage/ for the community of computational biologists to encourage its further development and/or its integration in general analytical workflows.

## Author contributions

PL and AR equally contributed to the conception, design and testing of the WG-Cluster algorithm; both the authors equally contributed also to the selection of the data and the case study for WG-Cluster application and to the interpretation of the algorithm output. Both the authors contributed to writing the manuscript.

### Conflict of interest statement

The authors declare that the research was conducted in the absence of any commercial or financial relationships that could be construed as a potential conflict of interest.
