# Supplementary material for: Detecting modules in biological networks by edge weight clustering and entropy significance
Source: Front Genet. 2015 Aug 27;6:265. doi: 10.3389/fgene.2015.00265 (PMC4551098; doi:10.3389/fgene.2015.00265)
Supplement: Supplementary file 1 [file Presentation1.PDF]

Supplementary Material: Detecting modules in biological networks by edge weight clustering and entropy significance

Paola Lecca [1,2] **and Angela Re** [3,*]

[1] *Centre for Integrative Biology, University of Trento, Italy*
[2] *Member of Association for Computing Machinery, New York, USA*
[3] *Laboratory of Translational Genomics, Centre for Integrative Biology, University of Trento, Povo (Trento), Italy*

Correspondence*:
Angela Re
Laboratory of Translational Genomics, Centre for Integrative Biology, University of Trento, via Sommarive 9, 38123 Povo (Trento),Italy, angela.re@unitn.it

1 SUPPLEMENTARY TABLES AND FIGURES

1.1 NETWORK CLUSTERING METHODS: EXPLORATORY ANALYSIS

An exploratory analysis was conducted preliminarily to the choice of the network edge weight based clustering algorithm. Here, we briefly report the results of that analysis motivating the selection of the K-means clustering methodology.

K-means refers to a specific algorithm that assigns labels to data points such that within-cluster variances are minimized. K-means is based on pairwise Euclidean distances between points, as the within-cluster variance (that is the sum of squared deviations from centroid) is equal to the sum of pairwise squared Euclidean distances divided by the number of points. A centroid is the multivariate mean in Euclidean space. Since non-Euclidean distances do not span Euclidean space, adopting non-Euclidean distance measures may prevent convergence of the K-means clustering.

A number of alternative clustering algorithms, partitioning ones and hierarchical ones, utilize similarities other than Euclidean (**Jay et al.** (2012); **Andreopoulos et al.** (2009)). Hierarchical clustering methods (both deterministic and stochastic) are not recommended to process data of omics size, considering the prohibitive computational times and RAM consumption. Due to these factors, we did not opt for any hierarchical clustering methods. Partitioning clustering methods are more efficient, and K-means is known to be one of the more efficient. K-means is linear in all relevant factors (iterations, number of clusters, and number of items to cluster). Furthermore, K-means is a non-model based network clustering methodology since it does not formalize a cluster as a parametrized model, and so has a special strength in clustering omics technology based networks where a priory knowledge is often not available.

As partitioning clustering methodologies alternative to K-means, we tested the Partitioning Around Medoids (PAM) and the Affinity Propagation (AP) methodologies. PAM clustering, differently from K-means, can be used in association with similarity measures other than the Euclidean one. However, the complexity of PAM is quadratic in the number of edges ($\mathcal{O}$(nr. of edges^2)), whereas the complexity of K-means is linear in the number of edges ($\mathcal{O}$(nr. of edges)). Since the network analyzed in this study consists of 5,769 nodes and 18,078 edges, the running times of PAM is almost 8 days on a Desktop PC with Windows 8.1, 3.01 GHz CPU and 16 Gb of RAM. K-means only employes 5.6 mins. Both the approaches return the same 29 sub-graphs (clusters) (see Supplementary Table 1).

Supplementary Table 1. Running times of K-means and PAM R implementation on the network case study (5,769 nodes and 18,078 edges) on Desktop PC, Windows 8.1 operating system, with a 64-bit quadri-core processor and 16 Gb of RAM.

Method	Time (secs)	Time (hours)	Number of clusters
K-means	336.36	0.0933	29
PAM (with Manhattan distance)	653,628.2	181.53 (7.5 days)	29
AP	587,52	163.2	3

The AP clustering approach has been recently applied in various fields, and, among others, bioinformatics is becoming increasingly important (**Frey and Dueck** (2007)). An advantage of AP clustering is in that it does not need to set the number of clusters whereas major disadvantages include poor efficiency and accuracy on large-scale data (**Liu et al.** (2013)) The time complexity of the AP clustering method is $\mathcal{O}$(nr. of edges^2) as for the PAM. The attempts of making the AP clustering method more efficient are promising but not consolidated yet (**Shanga et al.** (2012); **Shrivastava et al.** (2013)). With a running time of 7 days, AP clustering procedure implemented in R and based on the algorithms proposed by (**Frey and Dueck** (2007); **Bodenhofer et al.** (2011)) returned 3 sub-graphs yielding a Davies-Bouldin index close to

1, which is indicative of poor clustering quality. Note that the estimate of the optimal number of clusters by AP clustering is in disagreement with estimate consistently obtained by the K-means WCSS curve analysis (implemented in WG-Cluster), as well as by the Bayesian Inference Criterion for partitioning clustering (**Zhao et al.** (2008)) and Gap Statistic (**Tibshirani et al.** (2001)) methods (29 sub-graphs).

Our exploratory analysis permitted two conclusive observations: (1) the estimate of the optimal number of clusters, which is implemented in WG-Cluster, was confirmed by alternative criteria, and (2) network clusters detected by WG-Cluster are consistent in number and composition with those returned by alternative partitioning clustering methodologies. Notably those alternative approaches are characterized by neatly higher time complexity. Therefore, we finally opted for the K-means clustering method.

1.2 WG-CLUSTER TIME COMPLEXITY

We tested WG-Cluster performances on 20 random graphs generated by Erdos-Renyi algorithm with fixed edge probability (p= 8E-5) and increasing number of nodes. A relatively low network edge probability was selected to permit the generation of graphs whose time complexity was of the order of some days and whose data structure (vector-wise) allocation memory is lower than 2 Gb for R environment on 64-bit Windows 8.1 operating systems, we selected a relatively low edge probability. Processing times are reported in Supplementary Table 2. The first four reported processing times have been observed by running the code, whereas the remaining ones were estimated by the Eq. (1) that defines WG-Cluster time complexity TC as:

$$\begin{aligned} TC &= \mathcal{O}\big((N_{cl}^{(\text{max})}-1)\times NE\times n_{\text{iter}}\big)+\mathcal{O}\big(K\times NE\times n_{\text{iter}}\big)+\mathcal{O}\big(V\times(\log(V))^2\big) \\ &= t_u\Big[\big((N_{cl}^{(\text{max})}-1)\times NE\times n_{\text{iter}}\big)+\big(K\times NE\times n_{\text{iter}}\big)+\big(V\times(\log(V))^2\big)\Big] \end{aligned} \quad (1)$$

where $N_{cl}^{(\text{max})}$ is the maximum number of cluster (set to 250 in our case study), NE is the number of edges (18,078), n_{iter} is the K-means iterations (10,000), V is the number of vertices (=5,769), and t_u is the unit time step constant, that on 64-bit Windows 8.1 operating system with quadri-core processor and 16 Gb of RAM is about 5×10^{-7} secs.

1.3 NETWORK EDGES WEIGHTING SYSTEM

In the case study herein presented the edge weights of the network are the product of the differential co-expression score and the IntAct interaction score. This edge weight model is based on the rationale that the strength of the interaction between any two proteins in a certain condition depends on congruent protein expression and on the affinity by which the proteins interact with each other. In our application, we approximated protein expression by mRNA expression, by assuming that mRNA abundances are the main determinant of protein abundances, and we approximated the affinity of a protein-protein interaction by the corresponding IntAct score. The IntAct score, which ranges between 0 and 1, represents the reliability of the combined annotation evidences associated with a protein-protein interaction. By construction, the IntAct score aggregates experimental evidences throughout different biological contexts and it is not condition-specific; nonetheless we thought that it is strongly indicative of the affinity of a protein-protein interaction.

Furthermore, differential co-expression score and IntAct score are uncorrelated (Supplementary Figure 1). Since both scores could be informative and were found to be independent, we set out to integrate them in a multiplicative linear model to express the proportionality of the overall score of an edge to the differential co-expression score and to the IntAct score. The choice of this simple edge weight model was supported by comparing the clustering quality indices when we quantified an edge weight in the differential network only by the differential co-expression score or by the product of the two scores.

Supplementary Table 2. WG-Cluster processing times of Erdos-Renyi graphs with increasing numbers of nodes and edges on 64-bit Windows 8.1 operating system with quadricore processor and 16 Gb of RAM.

Nodes	Edges	Times (hours)	Times (days)
25,500	25,990	7.73	0.32
50,500	102,561	30.48	1.27
75,500	228,098	67.80	2.82
100,500	403,851	120.04	5.00
125,500	630,602	187.43	7.81
150,500	906,151	269.33	11.22
175,500	1,233,352	366.58	15.27
200,500	1,608,422	478.06	19.92
225,500	2,035,962	605.14	25.21
250,500	2,510,363	746.14	31.09
275,500	3,033,483	901.62	37.57
300,500	3,614,601	1,074.35	44.76
325,500	4,239,845	1,260.18	52.51
350,500	4,912,611	1,460.15	60.84
375,500	5,636,907	1,675.42	69.81
400,500	6,418,672	1,907.78	79.49
425,500	7,244,914	2,153.36	89.72
450,500	8,121,276	2,413.83	100.58
475,500	9,044,832	2,688.34	112.01
500,500	10,019,468	2,978.02	124.08

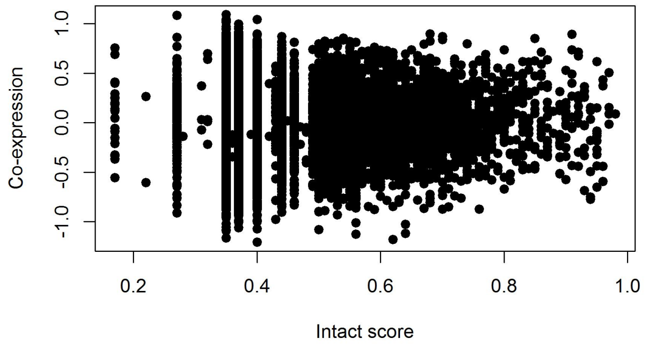

Supplementary Figure 1. Differential co-expression score versus IntAct affinity score scatterplot does not show any correlation pattern.

Indeed, the latter choice resulted in more compact and better separated clusters. In the next section, we show the cluster quality check performed on the differential network clustering.

1.4 CLUSTERING QUALITY CONTROL

We used the Davies-Bouldin index **Halkidi et al.** (2002); **Handl et al.** (2005); **Ristevski et al.** (2008); **Mittal et al.** (2014) as internal validation measure to estimate the quality of the clusters obtained with WG-Cluster. The internal validity measures are separation and compacteness. No external validation

methodology could be applied to our case study give the lack of any a priori knowledge of external information as class-label or ”purity” estimation of the clusters.

Let us denote by δ_k the mean distance of the points belonging to cluster C_k to their barycenter $G^{(k)}$:

$$\delta_k = \frac{1}{n_k} \sum_i ||M_i^{(k)} + G^{(k)}||.$$

For each pair of clusters k, k' ($k, k' = 1, 2, \ldots, K$), the maximum of the quantity

$$\frac{\delta_k + \delta_{k'}}{\Delta_{kk'}}$$

is calculated for all indices $k \neq k'$. $\Delta_{kk'}$ is the distance between the barycenters $G^{(k)}$ and $G^{(k')}$ of clusters C_k and $C_{k'}$:

$$\Delta_{kk'} = ||G^{(k')} - G^{(k)}||.$$

The Davies-Bouldin index is the mean value, among all the clusters, of the quantities M_k:

$$DB = \frac{1}{K} \sum_{k=1}^{K} M_k \max_{k' \neq k} \left(\frac{\delta_k + \delta k'}{\Delta_{kk'}} \right) \tag{2}$$

where $\max_{k' \neq k} \left(\frac{\delta_k + \delta k'}{\Delta_{kk'}} \right)$ is termed *maximal DB index*, and is usually denoted with R. $DB \in [0, +\infty)$ is a function of the ratio of the within-clusters scatter over the between-clusters separation. Since clusters are required to be compact and separated, lower Davies-Bouldin indices reflect better clustering configurations.

As shown in Supplementary Figure 2, the proportion of sub-graphs with a DB index between 0.55 and 0.65 is greater in the case where edge weights are the product of differential co-expression score and IntAct score compared to the case where edge weights are defined only by the differential co-expression. Furthermore, it is interesting to note that no sub-graph with a DB index ranging between 0.70 ad 0.75 was observed when we adopted the compound score instead of the differential co-expression score.

The different skewness of the two histograms reflects these differences. To test the significance of this skewness difference between two distributions we performed a single-sample Wilcoxon test on the absolute value of the skewness differences obtained from 1,000 random permutations of the differential co-expression scores sample and the differential co-expression × IntAct score sample. The test rejects the null hypothesis that the difference μ between the mean values of the skewness in the two samples is null (Wilcoxon statistics W = 500,500 and p-value less that 2.2e-16). Skewness values and Wilcoxon statistics are reported in Supplementary Table 3.

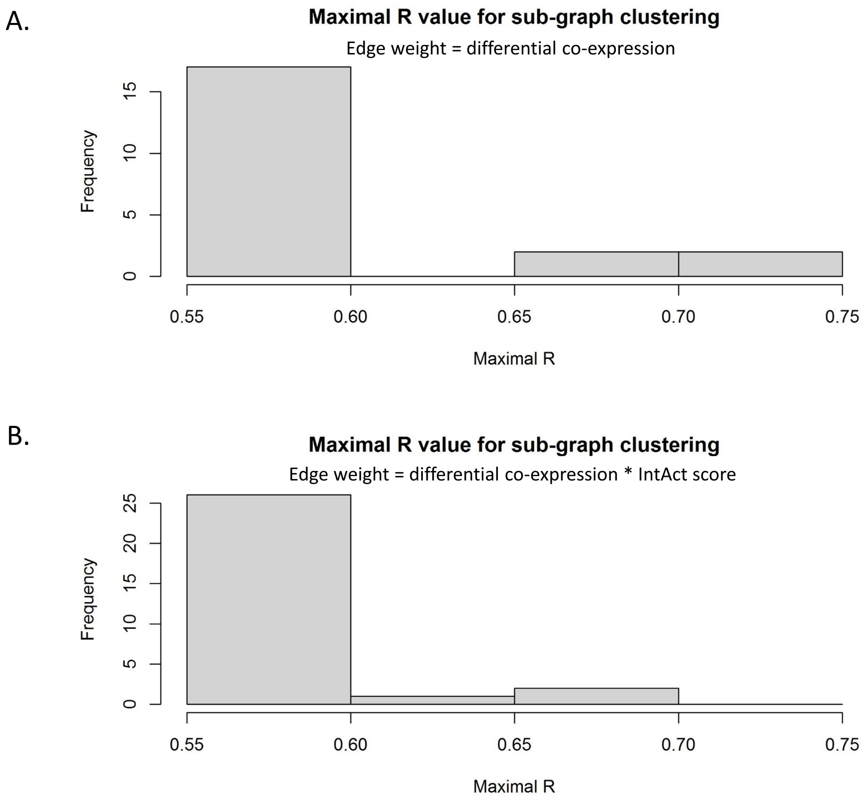

Supplementary Figure 2. Distributions of the DB index of the clusters obtained by weighting network edges by differential co-expression score (A), or by the product of differential co-expression and the IntAct scores (B).

Supplementary Table 3. WG-Cluster processing times of Erdos-Renyi graphs with increasing numbers of nodes and edges on 64-bit Windows 8.1 operating system with quadricore processor and 16 Gb of RAM.

Sample	Skewness
Differential co-expression score	1.607824
Differential co-expression × IntAct score	2.132293
Wilcoxon signed rank test with continuity correction<br>Null hypothesis $(H_0) : \mu = 0$<br><br>H_0 is rejected with W = 500,500 and p-value $<$ 2.2e-16.	

REFERENCES

Andreopoulos, B., An, A., Wang, X., and Schroeder, M. (2009), A roadmap of clustering algorithms: finding a match for a biomedical application, *Brief Bioinform*, 10, 3, 297–314, doi:10.1093/bib/bbn058

Bodenhofer, U., Kothmeier, A., and Hochreiter, S. (2011), Apcluster: an r package for affinity propagation clustering, *Bioinformatics*, 27, 17, 2463–2464, doi:10.1093/bioinformatics/btr406

Frey, B. J. and Dueck, D. (2007), Clustering by passing messages between data points, *Science*, 315, 5814, 972–976, doi:10.1126/science.1136800

Halkidi, M., Batistakis, Y., and Vazirgiannis, M. (2002), Clustering validity checking methods, *SIGMOD Rec.*, 31, 3, 19–27, doi:10.1145/601858.601862

Handl, J., Knowles, J., and Kell, D. B. (2005), Computational cluster validation in post-genomic data analysis, *BMC Bioinformatics*, 21, 15, 3201–3212, doi:10.1186/1471-2105-13-S10-S7

Jay, J. J., Eblen, J. D., Zhang, Y., Benson, M., Perkins, A. D., and et al. (2012), A systematic comparison of genome-scale clustering algorithms, *BMC Bioinformatics*, 13, Suppl 10, S7, doi:10.1186/1471-2105-13-S10-S7

Liu, X., Yin, M., Luo, J., and Chen, W. (2013), An improved affinity propagation clustering algorithm for large-scale data sets, *Ninth International Conference on Natural Computation (ICNC)*, 894–899

Mittal, M., R.K.Sharma, and V.P.Singh (2014), Validation of k-means and threshold based clustering method, *International Journal of Advancements in Technology*, 5, 2, 153–160, doi:10.1109/ICBBE.2008.143

Ristevski, B., Loshkovska, S., Dzeroski, S., and Slavkov, I. (2008), A comparison of validation indices for evaluation of clustering results of dna microarray data, *The 2nd International Conference on Bioinformatics and Biomedical Engineering, 2008.*, 21, 15, 587–591, doi:10.1109/ICBBE.2008.143

Shanga, F., Jiaoa, L., Shia, J., Wangb, F., and Gonga, M. (2012), Fast affinity propagation clustering: A multilevel approach, *Pattern Recognition*, 45, 1, 478–486, doi:10.1016/j.patcog.2011.04.032

Shrivastava, S. K., Rana, J., and Jain, R. (2013), Fast affinity propagation clustering based on machine learning, *International Journal of Computer Science Issues*, 10, 1, 302–309

Tibshirani, R., Walther, G., and Hastie, T. (2001), Estimating the number of clusters in a data set via a gap statistics, *J. R. Statist. Soc. B*, 69, Part 2, 411–423

Zhao, Q., Hautamaki, V., and Franti, P. (2008), Knee point detection in bic for detecting the number of clusters, *Advanced Concepts for Intelligent Vision Systems, Lecture Notes in Computer Science*, 5259, Part 2, 664–673, doi:10.1007/978-3-540-88458-3
